# Adverse Cardiovascular Effects of Anti-COVID-19 Drugs

**DOI:** 10.3389/fphar.2021.699949

**Published:** 2021-08-25

**Authors:** Dongling Liu, Xiang Zeng, Zufeng Ding, Fenghua Lv, Jawahar L. Mehta, Xianwei Wang

**Affiliations:** ^1^ Henan Key Laboratory of Medical Tissue Regeneration, Xinxiang Medical University, Xinxiang, China; ^2^ Laboratory of Environmental Medicine and Developmental Toxicology, Guangdong Key Laboratory of Environmental Pollution and Health, School of Environment, Jinan University, Guangzhou, China; ^3^ Division of Cardiology, University of Arkansas for Medical Sciences and Central Arkansas Veterans Healthcare System, Little Rock, AR, United States; ^4^ Department of Cardiology, Xinxiang Medical University First Affiliated Hospital, Weihui, China

**Keywords:** COVID-19, SARS-CoV-2, anti-COVID-19 therapy, cardiovascular complications, inflammation

## Abstract

Severe acute respiratory syndrome coronavirus 2 (SARS-CoV-2) or COVID-19 infection is the cause of the ongoing global pandemic. Mortality from COVID-19 infection is particularly high in patients with cardiovascular diseases. In addition, COVID-19 patients with preexisting cardiovascular comorbidities have a higher risk of death. Main cardiovascular complications of COVID-19 are myocardial infarction, myocarditis, acute myocardial injury, arrhythmias, heart failure, stroke, and venous thromboembolism. Therapeutic interventions in terms of drugs for COVID-19 have many cardiac adverse effects. Here, we review the relative therapeutic efficacy and adverse effects of anti-COVID-19 drugs.

## Introduction

Coronavirus disease 2019 (COVID-19) is a current global, emerging, and pandemic respiratory disease caused by an infection of SARS-CoV-2 (Huang et al., 2020). SARS-CoV-2 is a kind of RNA virus with a characteristic envelope and a linear single positive strand genome, which is different from severe acute respiratory syndrome (SARS) and Middle East respiratory syndrome (MERS) in infectivity and gene structure (Chen et al., 2020; Rabaan et al., 2020; Shereen et al., 2020). So far, there were more than 183 million documented global cases of COVID-19 and 4 million deaths according to reports of the World Health Organization (WHO) and Johns Hopkins University (Johns Hopkins University, 2021; WHO, 2021). In other words, the global pandemic of COVID-19 infection is still serious, although vaccines from different countries and companies have been used worldwide (Dong et al., 2020; Kissler et al., 2020; Zhang et al., 2020a). Therefore, it is necessary to discuss the adverse effects of different therapies.

So far, the main anti-COVID-19 therapies are drug treatments. However, there are few effective drugs, and cardiovascular complications of drugs and SARS-CoV-2 mutation make it harder for potential drugs to effectively control the COVID-19 pandemic (Hamid et al., 2020).

**FIGURE 1:**
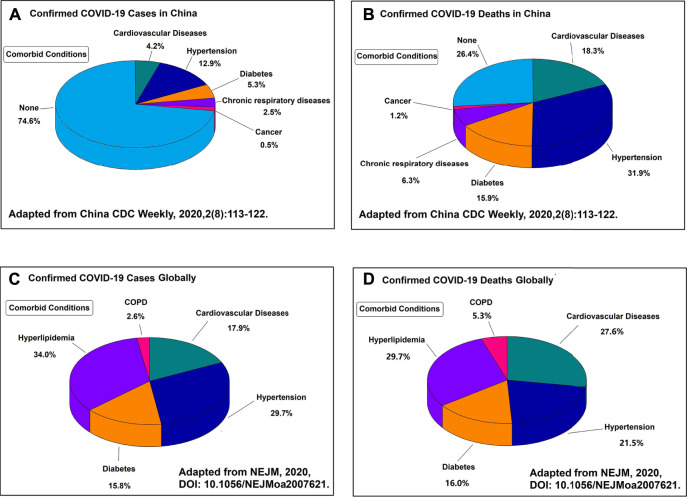
**(A)** Proportion of the Chinese patients and their comorbid conditions that were diagnosed with COVID-19. **(B)** Proportion of the Chinese patients and their comorbid conditions that died from COVID-19. **(C)** Proportion of the global patients and their comorbid conditions that were diagnosed with COVID-19. **(D)** Proportion of the global patients and their comorbid conditions that died from COVID-19. All the four figures indicate that the major complications of COVID-19 are the cardiovascular related diseases.

COVID-19 is more than just a lung disease, and cardiovascular complications are common and serious in patients with COVID-19 (Bikdeli et al., 2020; Lala et al., 2020; Lang et al., 2020; Li et al., 2020; Liu et al., 2020d; Madjid et al., 2020). COVID-19 patients with cardiovascular and respiratory comorbidities contribute to the highest case fatality rate (Figure 1) (CDC, 2020a; Clerkin et al., 2020; Giustino et al., 2020; Kunutsor and Laukkanen, 2020; Li et al., 2020a; Qiu et al., 2020). Cardiovascular disease patients are more susceptible to SARS-CoV-2 and more likely to develop severe COVID-19 (Akhmerov and Marbán, 2020; Chung et al., 2020; Driggin et al., 2020; Han, 2020; Katz et al., 2020; Mahmud et al., 2020). In particular, children with COVID-19 have also been reported to develop hyperinflammatory shock with characteristics similar to Kawasaki disease, including abnormalities of the coronary vessels and cardiac dysfunction (Akca et al., 2020; Alizargar, 2020; Toubiana et al., 2020). Common cardiovascular complications of COVID-19 are arrhythmias, direct cardiac injury, fulminant myocarditis, acute myocardial infarction, heart failure, pulmonary embolism, and disseminated intravascular coagulation (Aid et al., 2020; Barison et al., 2020; Guzik et al., 2020; Long et al., 2020; Maleszewski et al., 2020; Matsushita et al., 2020). Cytokine storm is associated with the severity and mortality of COVID-19 patients, which may be regulated by a key inflammation regulator known as the NLRP3 inflammasome (Liu et al., 2018; Liu et al., 2020a; Ruscitti et al., 2020; Ye et al., 2020). Excess activation of NLRP3 inflammasome increases the cardiovascular complication in COVID-19 patients (Bertocchi et al., 2020; Freeman and Swartz, 2020; Paniri and Akhavan-Niaki, 2020). However, to date, little is known about the cardiovascular complications of anti-COVID-19 drug therapy.

Current common anti-COVID-19 therapies include antiviral treatment, immunotherapy, convalescent plasma, traditional Chinese medicine, oxygen therapy, nutritional support, mechanical ventilation, and nursing care (CDC, 2020b; Inglis et al., 2020; Zhang, 2020g; Nih, 2021; Yetman and Vinetz, 2021). It is important to understand in-depth the cardiovascular complications of anti-COVID-19 therapy to further define the benefit of drug treatment strategies. Here, we summarize the cardiovascular complications of anti-COVID-19 drugs and discuss the performance of various treatment options to shed light on drug therapies for COVID-19.

## Mechanisms of Cardiovascular Complications of COVID-19

**FIGURE 2:**
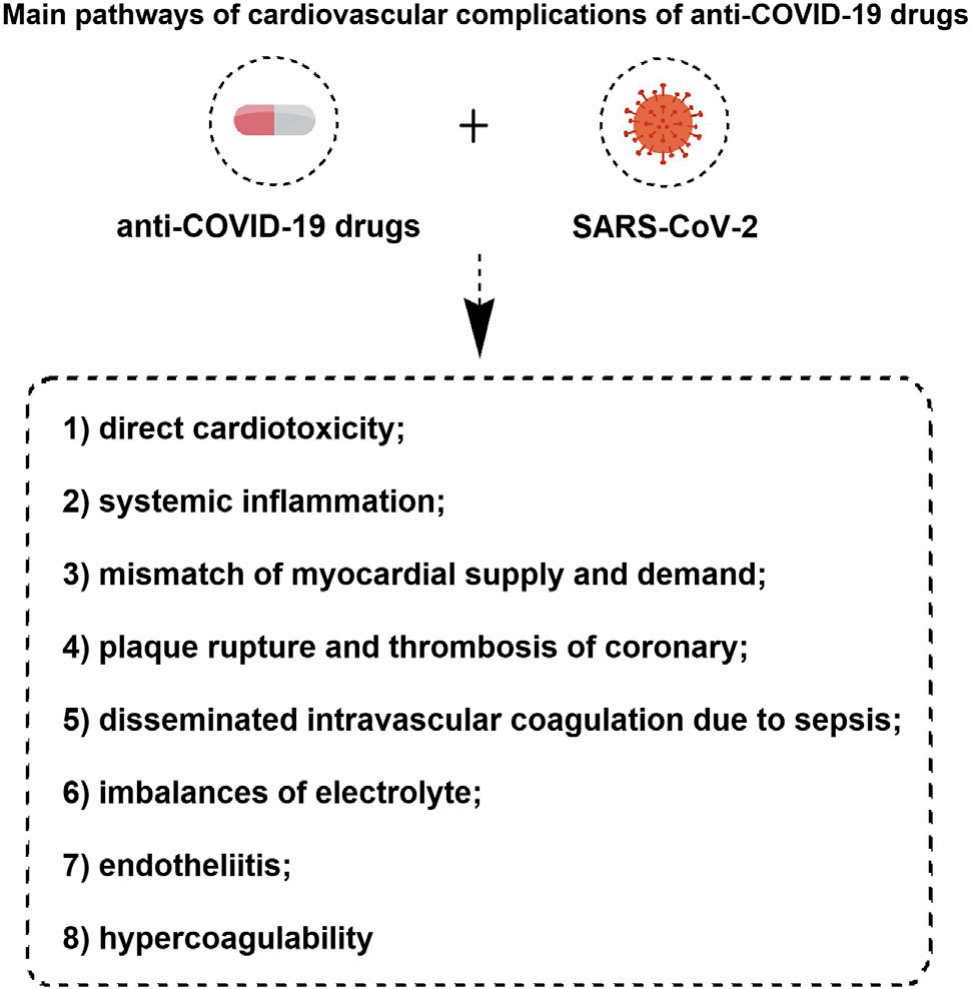
The main pathways of cardiovascular complications of anti-COVID-19 drugs.

The interplay between cardiovascular disease (CVD) and COVID-19 may manifest itself in three patterns: 1) CVD increases mortality among COVID-19 patients; 2) SARS-CoV-2 infection causes new CVD; 3) Drugs for CVD patients interfere with the physiology, pathophysiology and pharmacology of COVID-19 and vice versa (Ferrari et al., 2020). The mechanisms for the cardiovascular complications of COVID-19 are still not fully understood. However, the main pathways of cardiovascular complications of anti-COVID-19 drugs have been proposed are listed as follows (Figure 2): 1) direct cardiotoxicity; 2) systemic inflammation; 3) mismatch of myocardial supply and demand; 4) plaque rupture and thrombosis of coronary; 5) disseminated intravascular coagulation due to sepsis; 6) imbalances of electrolyte; 7) endotheliitis and 8) hypercoagulability (Bansal, 2020; Kang et al., 2020; Nishiga et al., 2020; Nägele et al., 2020; Varga et al., 2020).

**FIGURE 3:**
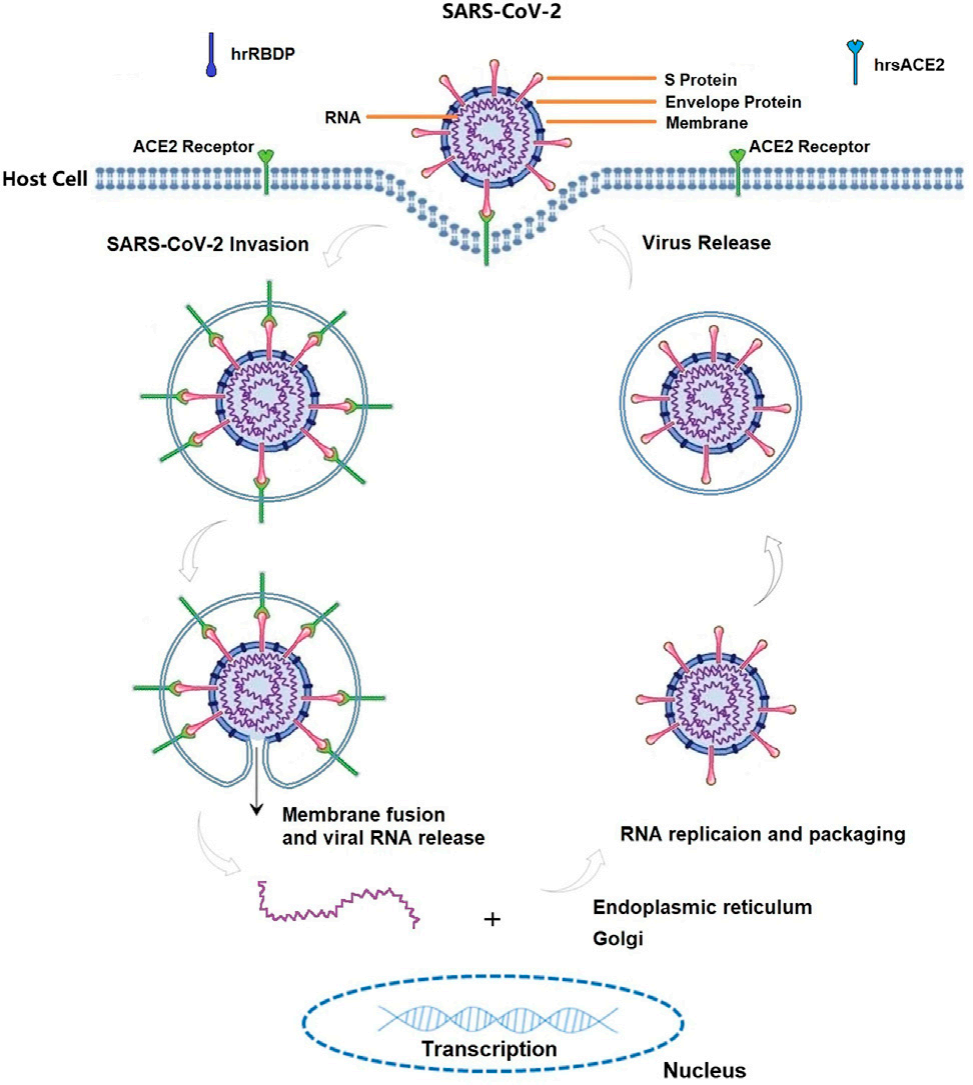
The life cycle of SARS-CoV-2 in host cells, annotated with the known mechanisms by ACE2 biology pathway. ACE2 can be recognized by the spike (S) protein on the surface of SARS-CoV-2, and their combination enable the virus to enter human cells in term of attachment, fusion and entry. S protein compose of S1 subunit (containing receptor-binding domain (RBD) which can bind to host receptor) and S2 subunit (fusing the viral and host membranes). Therefore, both ACE2 and S protein can serve as targets for the development of entry inhibitors, antibodies, and vaccines. Any substances modifying ACE2-S protein complex are valid targets for anti-COVID-19 agents. For instances, human recombinant soluble ACE2 (hrsACE2, its structure similar with ACE2) and recombinant RBD protein (hrRBDP, its structure similar with S protein) are competitively binding to S protein and ACE2 receptor, which ultimately participate in the fight against COVID-19.

There is a common consensus that receptor-mediated endocytosis is the main way for the virus to enter cells. Up to now, a well-known molecular mechanism of COVID-19 is that SARS-CoV-2 can directly or indirectly invade humans by binding to angiotensin converting enzyme 2 [ACE2, an important component of the renin-angiotensin-aldosterone system (RAS)], which can lead to alterations in the ACE2 signaling pathway and subsequently induce lung and myocardial injury (Figure 3) (Liu et al., 2020g; Brojakowska et al., 2020; Sharma, 2020; Wang et al., 2020b; Wang et al., 2020d; Zheng et al., 2020; Zhou et al., 2020). The interaction of SARS-CoV-2 with RAS leads to electrolyte imbalance and results in hypokalemia (Lumpuy-Castillo et al., 2020). As RAS inhibitors, angiotensin converting enzyme inhibitors (ACEIs), angiotensin receptor blockers (ARBs), and statins were used to decrease the risk of COVID-19 infection or severity by improving endothelial dysfunction (Liu et al., 2020f; De Spiegeleer et al., 2020; Lee et al., 2020; Nägele et al., 2020). Although some studies challenged ACEIs/ARBs/statin therapy for COVID-19, ACEIs/ARBs/statin treatment should continue in patients with COVID-19 based on available reported evidence (Liu et al., 2020d; Fosbol et al., 2020; Hasan et al., 2020; Mehta et al., 2020; Zhang et al., 2020d; Zhang et al., 2020e; Tetlow et al., 2021).

The most severe cases of COVID-19 are characterized by an acute systemic inflammatory response, cytokine storm, endotheliitis, and hypercoagulability. Systemic infection, coupled with hypoxia, can lead to imbalance/mismatch of the myocardial supply and demand ratio. Increased of systemic inflammation and shear stress due to elevated coronary blood flow causes plaque rupture and subsequent arrhythmias, acute myocardial infarction, and heart failure. It is well known that endothelial cells play a crucial role in the maintenance and regulation of vascular homeostasis and coagulation. Endodermatitis, characterized by endothelial dysfunction and hypercoagulability, is a common feature of major comorbidities that increase the risk of severe COVID-19 (Becker, 2020; Fan et al., 2020; Panigada et al., 2020).

## Treatment Plan for Cardiovascular Complications of COVID-19

Common treatment of COVID-19 patients with cardiovascular complications includes traditional care, coronary angiography and percutaneous coronary intervention if indicated, the use of anticoagulants and antiplatelet agents, and supportive care. Some patients with circulatory collapse may need extracorporeal circulatory support. Next, we briefly summarize the cardiovascular complications of anti-COVID-19 drug therapy (Table 1).

**TABLE 1:** Antiviral and anti-inflammatory drugs that have been suggested for the treatment of COVID-19 infection.

Antiviral and anti-inflammatory drugs	Targeted virions	Mechanism	Total direction of effect	Pharmacokinetic role (inhibitor or substrate)	Adverse effects	Administration method and dosage	References
Lopinavir/Ritonavir	SARS-CoV-2	Protease inhibitor, inhibits 3CLpro	No effect	An inhibitor for CYP3A4	Hypertension, prolonged P-R and prolonged QT interval, torsade de pointes, severe conduction disorders, and cardiac arrhythmias	Oral. Twice a day and two capsules each time with 200 mg/50 mg/capsule for adults. It is worth note that the course of treatment is not more than 10 days	Cao et al. (2020), Gerard et al. (2020), Li and De Clercq, (2020), Pillaiyar et al. (2020), Zhang et al. (2020g)
Remdesivir	SARS-CoV-2	Protease inhibitor, inhibits SARS-CoV-2 RNA-dependent RNA polymerase	Positive	An inhibitor for viral RNA polymerase	Hypotension, bradycardia, QTc prolongation, T-wave abnormality	Intravenous infusion. 200 mg on the first day, and followed by 100 mg on day 2–10 or until discharge	Beigel et al. (2020), Fan et al. (2020), Gubitosa et al. (2020), Gupta et al. (2020), Horby et al. (2020), Spinner et al. (2020), Touafchia et al. (2021), Wang et al. (2020g)
Interferon α	SARS-CoV-2	Protease inhibitor, viral load reduction through inhibition of replication	Positive	An inhibitor for CYP3A4	Ischemic cardiomyopathy, arrhythmias, and hypertension or hypotension	Aerosol inhalation. 5 million units or equivalent mixed with 2 ml of sterile water twice per day for adults	Hariramnile et al. (2020), Mantlo et al. (2020), Sallard et al. (2020), Wang and Fish (2020a)
Ribavirin	SARS-CoV-2	Viral load reduction through inhibition of replication	Negative	A substrate for viral RNA capping enzyme	Bradycardia, cardiac dysfunction, anemia, hypomagnesemia, dyspnea and chest pain; mitochondrial toxicity and energy metabolism disorder of cardiomyocytes	Intravenous infusion. 500 mg/time, twice or triple per day with a combination of interferon or lopinavir/ritonavir, and the course of therapy is not more than 10 days	Elfiky, (2020a), Elfiky, (2020b), Hung et al. (2020), Li and De Clercq, (2020), Ma et al. (2020), Wang et al. (2020c)
Chloroquine	SARS-CoV-2	Viral load reduction through inhibition of replication	Negative	An inhibitor for phosphatidylinositol-3-kinase (PI3K)/Akt pathway	Hypotension, hypokalemia, QRS and QT prolongation, atrioventricular block, arrhythmias and even coma	Oral. (1). 500 mg/time, twice per day, 7-days course for weight of patients over 50 kg; (2). 500 mg/time, twice per day in the first 2 days, and 500 mg/time, once per day in the next 5 days for weight of patients less than 50 kg. It is forbidden in patients with heart disease	Aggarwal et al. (2020), Beigel et al. (2020), Brown et al. (2020), Cavalcanti et al. (2020), Cui et al. (2020), Ferner and Aronson, (2020), Geleris et al. (2020), Jankelson et al. (2020), Ledford, (2020), Liu et al. (2020b), Mercuro et al. (2020), Rome and Avorn, (2020), Rosenberg et al. (2020), Shah et al. (2020), Wang et al. (2020c), Wong, (2020), Wu et al. (2020), Zhang et al. (2020g)
Baricitinib	SARS-CoV-2	Protease inhibitor, inhibits Janus kinase 1/2	Positive	An inhibitor of Janus kinases (JAK)	Hyperglycaemia, infections and thromboembolic events	Oral or a nasogastric tube. 4 mg (two 2-mg tablets) once daily for up to 14 days or until hospital discharge. It is recommended in combination with remdesivir in patients with COVID-19 who require oxygen or ventilatory support	Cantini et al., 2020, Favalli et al. (2020), Kalil et al. (2021), Magro, (2020), Richardson et al. (2020), Stebbing et al. (2020), Zhang et al. (2020f)
Arbidol	SARS-CoV-2	Protease inhibitor, inhibits glycoprotein	Positive	An inhibitor of SARS-CoV-2	Nausea, diarrhea, dizziness, liver injury, vomiting, and bradycardia	Oral. 200 mg/time and triple per day, and the course of therapy is not more than 10 days	Deng et al. (2020), Li et al. (2020), Vankadari, (2020), Wang et al. (2020e), Xu et al. (2020), Zhu et al. (2020)
Dexamethasone	SARS-CoV-2	Combat cytokine storm by limiting the production of and damaging effect of the cytokines	Positive	A substrate for CYP3A4	Arrhythmias, headache, agitation, dizziness, and increased appetite	Oral or intravenous infusion. 6 mg once daily for up to 10 days or until hospital discharge	Ahmed and Hassan, (2020), Garibaldi et al. (2020), Horby et al. (2020), Jo et al. (2021), Johnson and Vinetz, (2020), Lester et al. (2020), Lim and Pranata, (2020), Matthay and Thompson, (2020), Patel et al. (2020), Prescott and Rice, (2020), Sharun et al. (2020), Sterne et al. (2020), Vetter et al. (2020)
Tocilizumab	SARS-CoV-2	Inhibit the activity of IL-6 receptor, block the “cytokine storm” caused by IL-6 pathway	Positive	An inhibitor for Interleukin-6 (IL-6) receptor	cardiomyopathy, liver injury, and infection such as an increase in serum cholesterol, ALT, AST, and injection site reaction	Intravenous infusion. 400 mg dissolving 100 ml 0.9% sodium chloride; infusion time should be more than 1 h; the cumulative number of times of administration is 2 at most; caution with allergic reaction; forbidden for those with tuberculosis infection	Alattar et al. (2020), Brown et al. (2020), Fu et al. (2020), Hermine et al. (2021), Khiali et al. (2020), Luo et al. (2020), Morena et al. (2020), Muhovic et al. (2020), Xu et al. (2020)
Anakinra	SARS-CoV-2	Protease inhibitor, inhibits IL-1 receptor, block the “cytokine storm” caused by IL-1 pathway	Positive	An inhibitor of SARS-CoV-2	Hypersensitivities, hyperinfammation, breathing problem, nausea, vomiting diarrhea, headache, joint pain	Subcutaneous injection.100 mg twice a day for 72 h, then 100 mg daily for 7 days	Aouba et al. (2020), Bilia et al. (2020), Bozzi et al. (2021), Cauchois et al. (2020), Cavalli et al. (2020), Dos Santos, (2020), Haigh et al. (2020), Hossen et al. (2020), Huet et al. (2020), Jamilloux et al. (2020), Kooistra et al. (2020), Langer-Gould et al. (2020), Manjaly Thomas et al. (2020), Navarro-Millán et al. (2020), Pasin et al. (2021), Salvi and Patankar, (2020), Wu et al. (2020)
				An inhibitor for Interleukin-1 (IL-1) receptor			

### Antiviral Treatments

#### Lopinavir/Ritonavir

Lopinavir is a human immunodeficiency virus (HIV) protease inhibitor, which was approved by the US Food and Drug Administration (FDA) in 2000 and allowed for listing in China in 2008 (Oldfield and Plosker, 2006). Lopinavir inhibits the action of the enzyme 3-chymotrypsin-like protease and P-glycoprotein, which plays a crucial role in the distribution and elimination of lopinavir (Zhang et al., 2020c). Ritonavir is used in combination with lopinavir because it can increase the half-life of lopinavir by inhibiting the metabolizing enzyme cytochrome P450 3A (Abbvie, 2020). The combination of lopinavir and ritonavir has been preferred for the treatment of COVID-19 (Cao et al., 2020; Li and De Clercq, 2020; Pillaiyar et al., 2020). However, it has recently been shown to be ineffective according to a randomized controlled trial (Cao et al., 2020). Interestingly, common adverse reactions of the combination of lopinavir and ritonavir include hypertension, prolonged P-R and prolonged QT interval, torsade de pointes, severe conduction disorders, and cardiac arrhythmias (Badiou et al., 2003; Abbvie, 2012; Gerard et al., 2020). Additionally, the combination of lopinavir/ritonavir can induce drug-related cardiotoxicity by regulating cardiomyocyte pyroptosis, lysosome-mediated protein degradation, calcium signaling pathway, and the phosphatidylinositol 3-kinase (PI3K)/protein serine threonine kinase (Akt) signaling pathway (Reyskens et al., 2013). Therefore, changes in electrocardiograms and alteration of blood lipids should be monitored and checked in patients with COVID-19 and dyslipidemia. The current mode of administration of lopinavir/ritonavir is oral, and the recommended dose is twice a day and two capsules each time with 200 mg/50 mg/capsule for adults. The course of treatment lasts less than 10 days.

#### Remdesivir

Remdesivir is an inhibitor of viral RNA-dependent RNA polymerase (RdRp) with broad-spectrum activity against several members of the virus family such as coronaviruses (e.g., SARS-CoV and MERS-CoV) and filoviruses (e.g., Ebola). RdRp is essential for the replication of SARS-CoV-2 (Spinner et al., 2020). Remdesivir is currently the only drug approved by the FDA for the treatment of COVID-19. It is usually used in hospitalization of patients who require supplemental oxygen. Until now, remdesivir and dexamethasone have been recommended as first-line antiviral therapy for COVID-19 (Fan et al., 2020; Horby et al., 2020; Spinner et al., 2020). Remdesivir shows an excellent antiviral infection ability *in vivo* experiment (EC50 = 0.77 μM; CC50 > 100 μM; SI > 129.87) compared to favipiravir, ribavirin, penciclovir, nitazoxanide, nafamostat, and chloroquine (Wang et al., 2020c). Although remdesivir has a numerically faster time to clinical improvement than placebo in patients, there was no statistically significant difference between the two groups. Notably, remdesivir is not recommended for mild patients due to a high incidence of adverse events (Wang et al., 2020g). Another larger study confirms that remdesivir was superior to placebo in shortening recovery time and reducing the incidence of adverse events (Beigel et al., 2020). Known adverse events of remdesivir are hypotension, bradycardia, QTc prolongation, and T-wave abnormality (Gubitosa et al., 2020; Gupta et al., 2020; Touafchia et al., 2021).

#### Interferon-α (IFN-α)

IFN-α, a low molecular glycoprotein, is characterized by antiviral, antiproliferative, antidifferentiation, and immunoregulatory functions. IFN-α is used for the control of COVID-19 in light of the urgency to confront the COVID-19 pandemic (Hariramnile et al., 2020; Sallard et al., 2020; Wang and Fish, 2020a). Type I interferon inhibited SARS-CoV-2 *in vitro* experiments (Mantlo et al., 2020). The main adverse reactions of IFN-α include ischemic cardiomyopathy, arrhythmias, and hypertension or hypotension (Sleijfer et al., 2005; Pichler, 2006; Kuo et al., 2018). In addition, it causes endothelial dysfunction by promoting the expression of factors related to oxidative stress (Buie et al., 2017). IFN-α can also cause acute myocardial injury by enhancing atherosclerotic plaques polarization of in M1 macrophages and inhibit angiogenesis by regulating signal transduction and activator of transcription 1 (STAT1) and STAT3 (Nelson et al., 2015; Hsu et al., 2017). The mode of administration of IFN-α is aerosol inhalation, and the recommended dose is 5 million units or equivalent mixed with 2 ml of sterile water twice per day for adults. Although the drug is administered locally in the respiratory tract, one should be vigilant of its cardiotoxicity and pay close attention to electrocardiogram changes, the development of acute myocardial infarction, and heart failure, especially in elderly patients with preexisting cardiovascular diseases.

#### Ribavirin

Ribavirin is a purine nucleoside analogue with broad-spectrum antiviral activity, which can effectively inhibit the proliferation of a variety of respiratory viruses. Ribavirin overdoses can increase the risks of bradycardia (17%), anemia (27%), and hypomagnesemia (45%) (Muller et al., 2007). It is administered in different ways in various diseases (Ma et al., 2020). This drug can also be used in both pediatric and adult populations co-infected with hepatitis B and HIV. At present, ribavirin combined with interferon and/or lopinavir/ritonavir is recommended for patients with COVID-19 (Elfiky, 2020a; Elfiky, 2020b; Hung et al., 2020; Li and De Clercq, 2020). Wang et al. reported that ribavirin can inhibit virus growth (EC_50_ = 109.50 μM, CC_50_ > 400 μM, SI > 3.65) in Vero E6 cells (Wang et al., 2020c). Common adverse reactions of ribavirin include dyspnea and chest pain, especially in patients with COPD and asthma (Lu et al., 2015). Long-term (lasting 13 days) and high-dose (600 mg twice per day) ribavirin increased the risk of developing cardiovascular disease events and raised the risk of death in patients with cardiovascular disease (Beigel et al., 2017). Ribavirin can induce mitochondrial toxicity and energy metabolism disorders of cardiomyocytes by promoting mitochondrial calcium metabolism disorders (Lafeuillade et al., 2001). Therefore, ribavirin may increase the risk of cardiac dysfunction in patients with preexisting cardiovascular diseases. The recommended administration of ribavirin for COVID-19 is intravenous infusion (500 mg, 2–3 times a day) with interferon-α or lopinavir/ritonavir, and the duration of therapy is not longer than 10 days.

#### Chloroquine

Chloroquine is used mainly in the treatment of malarial and rheumatic diseases (Savarino et al., 2003; Wang et al., 2020c; Wu et al., 2020). It served to treat COVID-19 patients due to the characteristic of inhibiting endosomal acidification required for virus-host cell fusion. Chloroquine has been the drug of choice for large-scale use in the treatment of COVID-19 patients due to its wide availability, proven safety record, and relatively low cost (Cui et al., 2020; Ledford, 2020; Shah et al., 2020). However, excessive use of chloroquine can cause cardiovascular dysfunction such as hypotension, hypokalemia, QRS and QT prolongation, atrioventricular block, arrhythmias, and even coma (Wong, 2020). In addition, COVID-19 patients receiving a combination of hydroxychloroquine and azithromycin had a longer QT interval than those taking hydroxychloroquine alone in a cohort study (Mercuro et al., 2020). Furthermore, recent studies demonstrated that chloroquine and hydroxychloroquine were not effective drugs for COVID-19 (Brown et al., 2020; Cavalcanti et al., 2020; Ferner and Aronson, 2020; Rosenberg et al., 2020). The therapeutic dose of drugs is in a narrow concentration range that requires appropriate doses for different populations.

Common derivatives of chloroquine (CQ) are chloroquine phosphate (PCQ) and hydroxychloroquine (HCQ). PCQ, as an approved immune modulator, can effectively block SARS-CoV-2/2019-nCoV infection (EC_50_ = 1.13 μM; CC_50_ > 100 μM, SI > 88.50) in Vero E6 cells (Beigel et al., 2020). PCQ can control the electrophysiological activity of the heart by regulating the energy metabolism of cardiac myocytes, voltage-gated ion channels, calcium channel, and electrochemistry transporters (Mubagwa, 2020). However, CQ can induce serious cardiovascular reactions such as arrhythmias, shock, and Adams-Stokes syndrome (Pareek et al., 2018). Recently, several reviews have noted out that CQ and HCQ induce significant prolongation of the QT interval and potentially increase the risk of serious arrhythmias and sudden death (Aggarwal et al., 2020; Jankelson et al., 2020; Zhang et al., 2020g).

The structures and mechanisms of HCQ and CQ are similar to those of PCQ. Wang et al. showed that HCQ can effectively block the replication of SARS-CoV-2 *in vitro* and improve the clinical symptoms of patients with COVID-19 (Wang et al., 2020c). Previous studies have shown that HCQ can reduce cardiovascular risk, especially the incidence of cardiac dysfunction in patients with myocardial infarction (Liu et al., 2018; Rempenault et al., 2018; Schrezenmeier and Dörner, 2020). Although the FDA has authorized the use of HCQ and CQ for the treatment of COVID-19, one still needs to be aware of its adverse cardiovascular reactions such as atrioventricular block, cardiomyopathy, and heart failure. Compared with CQ, HCQ is less toxic and more potent in inhibiting SARS-CoV-2 infection *in vitro* (Liu et al., 2020b). There is no association between HCQ administration and the risk of composite endpoint of intubation or death based on an observational study (Geleris et al., 2020). There is no proven benefit for the use of HCQ in patients with COVID-19. In addition, it is worth noting that CQ has been revoked with the emergency use authorization (EUA) by the FDA (Rome and Avorn, 2020).

#### Baricitinib

Baricitinib is known as an inhibitor of Janus-associated kinase (JAK), specifically referring to JAK1 and JAK2, which is approved by the FDA and the EU for the treatment of rheumatoid arthritis with high efficacy and safety records (Markham, 2017). It was identified as the numb-associated kinase inhibitor (NAK) with selectivity of the adapter protein-2 complex (AP2)-associated protein kinase 1 (AAK1) and cyclin G-associated kinase (GAK) that are mediators or regulators of viral endocytosis (Stebbing et al., 2020). Inhibition of AAK1 by baricitinib can interrupt virus entry into cells and subsequently stop intracellular assembly and virus replication. In summary, baricitinib against COVID-19 relies mainly on inhibiting cytokine release and SARS-CoV-2 endocytosis (Magro, 2020; Richardson et al., 2020; Zhang et al., 2020f). A recent study found that baricitinib improved clinical conditions in 12 patients with mild and moderate COVID-19 (Cantini et al., 2020a). Another study demonstrated that baricitinib had a lower fatality rate and admission to the ICU, but a higher discharge rate compared with controls at week 1 and week 2 (Cantini et al., 2020b). However, baricitinib was not recommended to treat patients with severe COVID-19 with susceptible constitution, at least be cautious (Favalli et al., 2020). Up to now, baricitinib and remdesivir have been vapproved by the FDA through emergency use authorization (EUA) for hospitalized COVID-19 patients (Kalil et al., 2021). Nonetheless, baricitinib is not an approved standalone drug by the FDA for COVID-19 treatment. The common adverse effect of baricitinib on COVID-19 is hyperglycemia, infections, and thromboembolic events.

#### Arbidol

Arbidol is a non-nucleoside antiviral drug mainly for the treatment of influenza and other viral infections, which has inhibitory effects on both enveloped and nonenveloped viruses (Boriskin et al., 2008; Liu et al., 2009; Blaising et al., 2014). As a highly selective hemagglutinin inhibitor, arbidol can effectively target the hemagglutinin fusion machinery and prevent CoVs from anchoring the cell surface and invading cells (Blaising et al., 2014). Arbidol can block the trimerization of the SARS-CoV-2 spike glycoprotein and host cell adhesion, and effectively inhibit SARS-CoV-2 *in vitro* (Deng et al., 2020; Vankadari, 2020; Wang et al., 2020e). A recent study suggested that arbidol was superior to lopinavir/ritonavir against COVID-19 (Zhu et al., 2020). The combination of arbidol and lopinavir/ritonavir was better than lopinavir/ritonavir in anti-COVID-19 therapy (Deng et al., 2020). In addition, arbidol alone could be used to treat COVID-19 patients with mild symptoms (Xu et al., 2020). However, a recent study showed that neither arbidol nor lopinavir/ritonavir could beneficially affect the outcomes of COVID-19 patients, and contributed to adverse effects such as diarrhea, poor appetite, and abnormal liver function (Li et al., 2020b). Common cardiovascular adverse effects of arbidol include nausea, diarrhea, dizziness, liver injury, vomiting, and bradycardia. The recommended dose of arbidol for COVID-19 patients is 200 mg PO each time and three times per day, and the course of therapy is not longer than 10 days.

#### Other Antiviral Drugs

Other common candidate antiviral drugs for COVID-19 are fapiravir, penciclovir, sofosbuvir, and galidesivir (Pillaiyar et al., 2020; Richardson et al., 2020; Wang et al., 2020c). Their main adverse effects include hypotension and arrhythmias. Fapiravir is a broad-spectrum anti-influenza drug, which exerts an antiviral effect mainly by inhibiting RNA synthetase (Gao et al., 2020; Wang et al., 2020c). Sofosbuvir is an FDA-approved drug that is mainly used to treat patients with hepatitis C with various genotypes. Sofosbuvir is converted in a host cell to its active form, nucleoside triphosphate through phosphorylation, which terminates RNA replication in the nascent viral genome through competition with the nucleotides of invasive viruses (Sayad et al., 2020). Elfiky reported that sofosbuvir, ribavirin, remdesivir, galidesivir and tenofovir can be bound to COVID-19 RNA polymerase and are potent drugs against COVID-19 infection (Elfiky, 2020b). *In vitro* experiments indicate that remdesivir (EC_50_ = 0.77 μM; CC_50_ > 100 μM; SI > 129.87), fapiravir (EC_50_ = 61.88 μM, CC_50_ > 400 μM, SI > 6.46) and penciclovir (EC_50_ = 95.96 μM, CC_50_ > 400 μM, SI > 4.17) can effectively inhibit SARS-CoV-2 (Wang et al., 2020c). However, these drugs have their own cardiovascular risk. For example, Mulangu et al. (2019) reported that an Ebola patient who received remdesivir had hypotension and subsequently died due to cardiac arrest. Caldeira et al. (2018) suggested an increased risk of serious bradycardia among patients treated with sofosbuvir and amiodarone based on an observational study. Taken together, cardiovascular complications and risks of antiviral drugs need to be taken into account when used in COVID-19 patients.

### Anti-Inflammatory and Immunotherapy Drugs

Anti-inflammatory and immunotherapy drugs can alter the workings of the immune system, so it can find, attack, and eliminate invasive pathogens and finally get rid of them. Thus, it is an efficient therapeutic option against viral infections, including patients with COVID-19 characterized by the cytokine storm (Aminjafari and Ghasemi, 2020; Blanco-Melo et al., 2020; Vabret et al., 2020).

#### Dexamethasone

Dexamethasone, as a representative anti-inflammatory drug, is a broad-spectrum immunosuppressor approved by the FDA with high activity and a long duration of action. It will inhibit the release and subsequent detrimental effect of cytokines to further combat symptoms of hyperinflammation or cytokine storm in COVID-19 (Lim and Pranata, 2020; Sharun et al., 2020). Recently, preliminary clinical trials of dexamethasone have been approved by the WHO based on the significant improvement in reducing mortality by 35% in ventilated patients and 20% in other patients receiving oxygen (Horby et al., 2020; Patel et al., 2020; WHO, 2020). However, there is a lack of clinical benefit of dexamethasone in non-critical patients without respiratory support such as ventilation or oxygen (Johnson and Vinetz, 2020; Lester et al., 2020). Dexamethasone can increase mortality in patients without critical illness who did not receive respiratory support (Matthay and Thompson, 2020). It is notable that a low dose of dexamethasone could only reduce the mortality of patients with severe COVID-19, but had no effect on the mortality of patients with mild and moderate COVID-19 patients; while high doses of dexamethasone contributed to more harm than good (Ahmed and Hassan, 2020; Prescott and Rice, 2020; Sterne et al., 2020). Additionally, the combination of dexamethasone and remdesivir may reduce mortality, which has been recommended to treat COVID-19 patients with mild-moderate conditions (Garibaldi et al., 2020; Vetter et al., 2020; Jo et al., 2021). It is worth noting that dexamethasone treatment can lead to several adverse effects such as arrhythmias, headache, agitation, dizziness, and increased appetite.

#### Tocilizumab

Tocilizumab, a humanized recombinant monoclonal antibody against interleukin-6 (IL-6), can inhibit the activity of the IL-6 receptor by binding to its membrane-bound and soluble forms, and thus block the “cytokine storm” caused by IL-6 (Xie et al., 2019). Recently, tocilizumab was used to treat COVID-19 patients at risk of cytokine storm (Brown et al., 2020). However, the adverse effects of tocilizumab should not be ignored, such as cardiomyopathy and liver injury (Muhovic et al., 2020). Gabay et al. (2016) found that tocilizumab induced cardiomyocyte apoptosis and mitochondrial oxidative stress resulting in cardiotoxicity, manifested as hypertension, hypercholesterolemia and myocardial infarction, which remind clinicians to pay attention to the above-mentioned adverse effects of tocilizumab when combating COVID-19. The most common adverse reactions and events were infections that were characterized by an increase in serum cholesterol, ALT, AST, and injection site reactions (Khiali et al., 2020). Luo et al. (2020) found that tocilizumab alleviated inflammatory activity in COVID-19 patients. Xu et al. (2020) demonstrated that abnormally elevated C-reactive protein and the number of lymphocytes decreased in 84 and 37% of patients, respectively. The average discharge time of patients treated with tocilizumab was 15.1 days, and this therapy effectively improved mortality in patients with severe and critically ill COVID-19 (Xu et al., 2020). It is worth of mentioning that an increasing trend of IL-6 occurs immediately when tocilizumab usage is reduced. Alattar et al. (2020) showed that tocilizumab caused adverse events such as anemia, QT interval prolongation, and ananine aminotransferase increase. Morena et al. (2020) demonstrated that COVID-19 patients treated with tocilizumab experienced adverse events included thrombocytopenia, increase of hepatic enzymes, and serious bacterial and fungal infections. Hermine et al. (2021) reported that tocilizumab induced cardiovascular adverse events manifested by hypertension, anemia, lymphopenia, and neutropenia, and bacterial sepsis. Fu et al. (2020) pointed out that tocilizumab treatment inhibited the inflammatory storm, resulting in an improved clinical outcome. Castagné et al. (2019) found that tocilizumab was associated with an elevated cholesterol level, which decreased inflammatory proteins and restored the antiatherogenic function of HDL cholesterol. It should be used with caution in patients with dyslipidemia and hypertension. The recommended dose of tocilizumab for COVID-19 patients is 400 mg dissolved in 100 ml of 0.9% sodium chloride; infused in >1 h 2 times at most; while looking for an allergic reaction.

#### Anakinra

Anakinra is one of the anti-interleukin 1 antagonist which can inhibit actions of IL-1 receptor to suppress the pathogeny of proinflammatory cytokine storm in COVID-19 (Aouba et al., 2020; Dos Santos, 2020; Jamilloux et al., 2020; Salvi and Patankar, 2020; Wu et al., 2020). Therefore, it has been used for the treatment of COVID-19 patients. Several studies indicated that anakinra is a safe and effective drug combating COVID-19 patients with cytokine storm signs (Bilia et al., 2020; Cauchois et al., 2020; Haigh et al., 2020; Huet et al., 2020; Navarro-Millán et al., 2020; Bozzi et al., 2021; Pasin et al., 2021). Cavalli et al. (2020) found that high-dose anakinra was associated with clinical improvement in 72% of patients with COVID-19 and acute respiratory distress syndrome in a retrospective cohort study. Langer-Gould et al. (2020) demonstrated that early identification and treatment of COVID-19 cytokine storm with anakinra significantly improved outcomes after mechanical ventilation. Kooistra et al. (2020) showed that anakinra can reduce clinical symptoms of hyperinflammation in critically ill COVID-19 patients. The main adverse effects of anakinra were hypersensitivities, hyperinfammation, breathing problem, nausea, vomiting diarrhea, headache, and joint pain (Hossen et al., 2020; Kooistra et al., 2020; Manjaly Thomas et al., 2020).

### Convalescent Plasma for COVID-19 Treatment

Convalescent plasma refers to plasma collected from COVID-19 convalescent patients, which can contribute to a safe and effective therapy for COVID-19 patients. Previous studies in a variety of viral respiratory diseases, such as SARS and MERS, indicated that convalescent plasma can reduce mortality. Therefore, convalescent plasma may provide a potential therapy for COVID-19 patients (Bloch et al., 2020; Chen et al., 2020; Tiberghien et al., 2020). Notably, the efficacy of convalescent plasma against COVID-19 remains to be proved (Casadevall and Pirofski, 2020; Roback and Guarner, 2020).

Several observed studies found that plasma convalescent therapy is effective and specific for COVID-19 (Duan et al., 2020; Shen et al., 2020; Ye et al., 2020). However, there is one point of view that convalescent plasma is ineffective for COVID-19 (Pathak, 2020; Rogers et al., 2020). In a word, the efficacy of convalescent plasma therapy for COVID-19 is controversial. Adequately powered and randomized controlled trials are needed to confirm the efficacy of convalescent plasma therapy for COVID-19 (Bloch, 2020; Liu et al., 2020e). Recently, several randomized controlled trials demonstrated that the efficiency in different cases using plasma convalescent therapy is inconsistent (Agarwal et al., 2020; Li et al., 2020c; Klassen et al., 2020; Simonovich et al., 2021). However, convalescent plasma was found to be relatively safe, in more than 20,000 patients with a low incidence of severe adverse events (<1%) (Joyner et al., 2020). The general known cardiovascular risks of plasma transfusion generally include transfusion-associated circulatory overload, hypercoagulablility, and thrombosis (Sanfilippo et al., 2021).

### Traditional Chinese Medicine for COVID-19 Treatment

Traditional Chinese Medicine (TCM) is usually defined by a mixture of herbal plants or their extracts which comprise hundreds of constituents with various differing physiochemical properties (Zhou, 2020). TCM is a unique health resource in China, which is also a vast and large untapped resource in the world. For example, artemisinin, a safe and effective antimalarial drug, was originally extracted from Artemisia annua by TCM scientific workers (Tu, 2016). In addition, in view of the remarkable therapeutic effects that TCM achieved during the SARS epidemic in 2003, TCM raises hopes for the prevention and control of COVID-19 (Luo et al., 2020). Several TCMs have been recommended by the China National Health Commission to treat COVID-19 patients because the advantages far outweigh the disadvantages, which are characterized mainly by promoting neutrophil-mediated inflammation and reducing macrophage-mediated anti-inflammatory activity (Wang et al., 2020f; Tong et al., 2020; Zhang, 2020b). Over 90% of COVID-19 patients in China have been treated with TCM, and some studies showed relief of symptom, reducing the time of onset of fever and reducing the severity of the disease (Du et al., 2020; Liu et al., 2020c; Ren et al., 2020). Common TCMs combating COVID-19 are HuoxiangZhengqi capsules, LianhuaQingwen capsules, Jinhuaqinggan granula, ShufengJiedu capsules, Fangfengtongsheng pill, Qingfeipaidu decoction, Suhexiang pill, Angongniuhuang pill, and XueBijing injection (Li et al., 2020d; Ren et al., 2020; Tong et al., 2020; Wang et al., 2020f). Different of the above-mentioned TCMs are used to treat different diseases and different courses of the same disease (Table 2). The key mechanism of TCM is inflammation regulation (Li et al., 2020). In addition, TCM controls cardiovascular risk factors such as hypertension and diabetes (Hao et al., 2017). Although attention should be paid to the possible adverse effects of TCM, the benefits of TCM in the treatment of COVID-19 are also obvious (Gray and Belessis, 2020; An et al., 2021; Ren et al., 2021). So far, the integration of TCM and Western medicine is effective for COVID based on previous pharmacological studies (Chen and Chen, 2020; Ni et al., 2020; Xu and Zhang, 2020).

**TABLE 2:** Nine TCMs recommended by guidelines of treatment for COVID-19.

TCMs	Stage of diseases	Symptoms	References
HuoxiangZhengqi capsules (HXZQ) - 藿香正气胶囊	Medical observation period	Fatigue with gastro-intestinal discomfort	Luo et al. (2020), Tong et al. (2020), Wang et al. (2020f)
LianhuaQingwen capsules (LHQW) - 连花清瘟胶囊	Medical observation period	Fatigue with fever	Li et al. (2020), Luo et al. (2020)
			Tong et al. (2020), Wang et al. (2020f)
JinhuaQinggan granula (JHQG) - 金花清感颗粒	Medical observation period	Fatigue with fever	Wang et al. (2020f)
ShufengJiedu capsules (SFJD) - 疏风解毒胶囊	Medical observation period	Fatigue with fever	Tong et al. (2020), Wang et al. (2020f)
FangfengTongsheng pill (FFTS) - 防风通圣丸	Medical observation period	Fatigue with fever	Wang et al. (2020f)
QingfeiPaidu decoction (QFPD) - 清肺排毒汤	Clinical treatment period	Clinical treatment period mild, general, and severe cases	Du et al. (2020), Ren et al. (2020), Wang et al. (2020f), Zhang et al. (2020b)
Suhexiang pill (SHX) - 苏合香丸	Clinical treatment period	Clinical treatment period mild, general, and severe cases	Luo et al. (2020), Wang et al. (2020f)
AngongNiuhuang pill (AGNH) - 安宫牛黄丸	Clinical treatment period	Several cases and critical cases	Luo et al. (2020), Wang et al. (2020f)
Xuebijing injection (XBJ) - 血必净注射液	Clinical treatment period	Several cases and critical cases	Luo et al. (2020), Tong et al. (2020), Wang et al. (2020f)

### Effects of Combining Drug Therapy on Cardiovascular System

A combination of drugs can on one hand be beneficial and on the other hand harmful because of side effects. Lopinavir and ritonavir are metabolized by CYP3A and interact with a variety of antiarrhythmic drugs, such as amiodarone and digoxin, which can increase the risk of adverse cardiovascular events. Antiviral drugs such as ritonavir can interact with nonvitamin K-dependent oral antiplatelet agents such as clopidogrel. The combination of ribavirin and IFN-α may aggravate the dysfunction of vascular endothelial cells caused by the virus (Schmidt et al., 2020). Therefore, the combination of ribavirin and IFN-α is not recommended for the treatment of COVID-19 infection. Chloroquine is primarily metabolized by the kidney and should not be combined with inhibitors of CYP1A1, CYP2D6, and CYP3A4 such as lopinavir/ritonavir. There are drug interactions between arbidol and CYP3A4 inhibitors or inducers, and long-term use of arbidol and CYP3A4 inhibitors or inducers can lead to cardiac toxicity (Liu et al., 2009). Tocilizumab can alter lipid metabolism and should not be combined with lopinavir/ritonavir (Gabay et al., 2016). Generally, it is not recommended to use three or more antiviral drugs at the same time. In case of intolerable adverse effects, the relevant drugs should be stopped (NHC, 2020a).

### Other Treatments and Therapeutic Schedules

Inappropriate use of antibacterials, especially the combination of three or more broad-spectrum antibacterial drugs should be avoided in patients with COVID-19 infection because that there were many complications such as hypertension, hyperglycemia, fever, dry cough, and dyspnea (Yang et al., 2020). Although corticosteroids have the action to reduce the severity of myocarditis and cytokine storm in COVID-19 patients, they inhibit viral RNA clearance. Therefore, it recommends using COVID-19 patients with severe symptoms (Ling et al., 2020; Russell et al., 2020). In addition, the intravenous transplantation of mesenchymal stem cells (MSC) can improve the condition of patients with COVID-19, especially for critical cases (Leng et al., 2020). It specifically demonstrated that increased peripheral lymphocytes and IL-10, decreased C-reactive protein and TNF-α, and disappear of the overactivated cytokine-secreting immune cells, such as CXCR3+CD4+ T cells, CXCR3+CD8+ T cells, and CXCR3+ NK cells, after MSC treatment compared to those in the placebo control group. The principles of treatment in severe and critical cases include symptomatic treatment, prevention of complications and secondary infections, treatment of underlying diseases, and timely maintenance of organ function. The main measures of organ function support are respiratory support (oxygen therapy and mechanical ventilation), circulatory support (liquid balance strategy, improvement of blood circulation with hemodynamic monitoring), and renal replacement therapy in patients with renal dysfunction, plasma therapy from convalescent patients, and blood purification (plasma exchange, adsorption, perfusion, filtration) (NHC, 2020b).

## Perspective

Once COVID-19 patients are treated, medical personnel must pay attention to baseline cardiovascular health and adjust the treatment plan in time according to changes in heart rate, blood pressure, blood lipids, cardiac function, and electrocardiogram. Medical staff should also take note of drug-drug interactions to avoid drug-induced myocardial injury. In addition, indexes of myocardial injury and cardiac function should be monitored by combining laboratory and imaging results. Clinicians need to continue to assess the efficacy of combination drugs. Combining of three or more antiviral drugs is not recommended, especially in elderly patients. Although progress has been made in the search for drugs to treat infection, a reliable drug screening model *in vitro* and *in vivo* should be built with collaborative innovation.

## Abbreviations

ACE2, angiotensin-converting enzyme 2; CVD, Cardiovascular diseases; COVID-19, Coronavirus disease 2019; CQ, chloroquine; ECG, electrocardiogram; FDA, Food and Drug Administration; HCQ, hydroxychloroquine; HIV, human immunodeficiency virus; IFN-α, Interferon-α; MERS, Middle East respiratory syndrome; NHC, National Health Commission of the People’s Republic of China; PCQ, chloroquine phosphate; SARS, severe acute respiratory syndrome; SARS-CoV-2, Severe acute respiratory syndrome coronavirus 2; TCM, traditional Chinese medicine; WHO, World Health Organization.
